# One Year On: Evaluation of the Cambridgeshire and Peterborough Staff Mental Health Service, a Bespoke Mental Health Clinic for Healthcare Workers

**DOI:** 10.1192/bjo.2022.398

**Published:** 2022-06-20

**Authors:** Muzaffer Kaser, Theodora Karadaki, Zoe Martin, Cathy Walsh

**Affiliations:** 1 Cambridgeshire and Peterborough NHS Foundation Trust, Cambridge, United Kingdom; 2 Department of Psychiatry, University of Cambridge, Cambridge, United Kingdom

## Abstract

**Aims:**

The Staff Mental Health Service (SMHS) at the Cambridgeshire and Peterborough NHS Foundation Trust is a multidisciplinary team providing rapid access assessments and treatments to NHS staff in all roles. The service was launched in September 2020 and obtained recurrent funding in 2021. Previously we reported initial clinical findings from the service suggesting high rates of moderate to severe depression, anxiety, and post-traumatic stress symptoms (Kaser et al. 2021). In this report, we present the clinical and demographic findings, and post-treatment outcomes from the first-year evaluation of the SMHS.

**Methods:**

Demographic and clinical data were collected as part of service evaluation at the SMHS. Depression, anxiety, and posttraumatic stress symptoms were evaluated by Patient Health Questionnaire (PHQ-9), Generalised Anxiety Disorder Assessment (GAD-7), and Posttraumatic Symptom Checklist – Civilian Version (PCL-C). Non-parametric Wilcoxon rank test was used to compare pre- and post-treatment symptom scores.

**Results:**

The service received 515 referrals in the first year. 39.6% of the patients were off work at the time of the referral. 81.2% patients were female and 75.3% were of white ethnicity. Median time from referral to assessment was 14 days. According to the clinical data (n = 320), 85.3% of the patients had moderate to severe depressive symptoms (mean PHQ-9: 16.25 ± 6.1), and 80.9% of the patients had moderate to severe levels of anxiety symptoms (mean GAD-7: 13.62 ± 4.7). Staff patients endorsed high levels of traumatic stress with 82.5% scoring higher than the established cut-off (PCL-C > 14) (PCL-C mean score: 19.14 ± 5.7). Analyses from patients completing treatment at the service showed significant improvements in depression (Z=−3.38, p = 0.001), anxiety (Z= -4.09, p < 0.001), and PTSD symptoms (Z= -4.99, p < 0.001).

**Conclusion:**

The Staff Mental Health Service had a persistent noticeable demand in its first year corresponding to two percent of the total workforce of the local trusts. Healthcare workers presenting to the service had high rates of moderate to severe depression, anxiety, and PTSD symptoms. Multidisciplinary treatment at the SMHS led to significant improvements in psychiatric symptoms. A health economics analysis of the service is currently underway.

